# Human-Mouse Chimerism for Organogenesis Without Neural or Germline Contributions

**DOI:** 10.7150/ijbs.119609

**Published:** 2026-09-03

**Authors:** Sen Ye, Kailibinuer Kadier, Ye Yi, Guihui Qin, Borong Huang, Siyi Fu, Cantas Alev, Ren-He Xu

**Affiliations:** 1 Center of Reproduction, Development & Aging, Institute of Translational Medicine, Faculty of Medicine, and Ministry of Education Frontiers Science Center on Precision Oncology, University of Macau, Taipa, Macau SAR 999078, China.; 2 Institute for the Advanced Study of Human Biology (ASHBi), University of Kyoto, Kyoto, Japan.

**Keywords:** neural cells, germline cells, ethics, chimera, organogenesis

## Abstract

Human pluripotent cells, including embryonic stem cells (ESCs), have been used for interspecies blastocyst complementation, with the potential to generate human organs in animals for clinical applications. However, ethical concerns exist as human cells may contribute to the germline and central nervous system (CNS) in chimeric animals. To prevent such concerns, here we generated *BLIMP1* and *PAX6* knockout human ESCs (hESCs) that are capable of contributing to all cell and tissue types, except the germline and CNS in human-mouse chimeras. To overcome the low chimerism efficiency of hESCs, we also engineered *BLIMP1^-/-^*/*PAX6^-/-^* hESCs with a doxycycline-inducible system to express the anti-apoptotic gene *BCL2* (*iBCL2*) and injected them into *Igf1r^-/-^* mouse blastocysts, which remarkably enhanced the chimerism efficiency. To test organogenesis potential, mesenchymal stem cells were generated from *BLIMP1^-/-^*/*PAX6^-/-^*/*iBCL2^+^* hESCs injected into *Sox9^+/-^* mouse blastocysts, which rescued *Sox9* haploinsufficiency skeletal defects and contributed to mesenchymal tissues of the chimeric fetuses and neonates. Thus, we provide a simple and effective approach to generating human-animal chimeras that are free of ethical concerns.

## Introduction

Interspecies chimerism is one method to test the developmental potential of pluripotent stem cells (PSCs) by injecting the cells into a recipient blastocyst-stage or earlier embryo followed by uterine implantation into a surrogate 1. It has been demonstrated that human PSCs (hPSCs), including human embryonic stem cells (hESCs) and induced pluripotent stem cells (iPSCs), can contribute to all three germ layers of a chimeric embryo 2. Furthermore, blastocyst complementation has been attempted to generate human organs in chimeras by knocking out specific genes essential for the development of an organ of interest in the host. The first proof-of-concept was shown by injecting wild-type (WT) donor mouse ESCs into the mouse blastocyst with *RAG-2* knockout (KO), which led to development of only donor-derived B and T lymphocytes in the chimeras 3. Rat PSCs injected into mouse blastocyst with *Pdx1* KO (to disable pancreatogenesis) also generated functional rat pancreas in the chimeras 4. More recently, genetically modified hPSCs with enhanced viability for chimerism generated humanized skeletal muscles and mesonephros in porcine fetuses with multigene KO to create niches conducive to the development of these tissues and organs 5, 6.

However, the risk of germline or neural contributions by human cells has posed serious ethical concerns, restricting human-animal chimerism studies. As an example, a recent study demonstrated that transplanting human cortical organoids into the somatosensory cortex of immunocompromised rats allows the growth of mature human brain cells and even drives reward-seeking behavior in the chimeras 7. These findings potentially indicate that neural integration of human cells may cause human-like behavior or mindsets in chimeras, which could also be passed on transgenerationally via gametes. Thus, the International Society for Stem Cell Research guidelines urge researchers to prevent contribution of human cells to the central nervous systems (CNS) and germlines of the chimeras and prohibit production of chimeric gametes 8. However, there also appears to be no clear regulations for the end point of chimeric studies as it depends on ethical, regulatory, and scientific considerations. Ethics committees usually recommend terminating human-mouse chimera experiments before birth and terminate human-animal chimera experiments involving larger animals at an early stage of pregnancy to avoid the above concerns 9. However, such restrictions prevent the proper maturation of human organs in chimeras, thereby limiting the utility of such studies to understanding the full developmental potential of human cells in animal fetuses and neonates.

One strategy to overcome such restrictions is to delete key genes required for the proper differentiation of hPSCs into germline and neural cells, to circumvent the contribution of human cells to those tissues in chimeric animals. Hashimoto *et al.* proved this strategy by knocking out *Prdm14* and *Otx2* in donor mouse ESCs followed by chimerization with recipient mouse blastocysts 10. However, both genes do not execute exactly the same functions in humans 11-13. For human organogenesis through human-animal chimerism, it remains essential to identify the genes critical for human germline and neural development, so that they can be selectively disrupted in donor hPSCs. Here, we selected and knocked out B lymphocyte-induced maturation protein 1 (*BLIMP1*) and Paired box protein 6 (*PAX6*) in hPSCs, which were then injected into the mouse blastocysts to generate human-mouse chimeras. We show that the double knockout (DKO) of *BLIMP1* and *PAX6* is sufficient to prevent germline and neural contributions of hESCs — while retaining their developmental capacity to generate various cell types of tissues including that of bone and cartilage — in chimeric mice. Hence, we provide an effective strategy to generate ethically-acceptable human-animal chimeras for basic and translational research.

## Results

### *BLIMP1* KO in hESCs prevents germline differentiation

To identify a key determinant for primordial germ cell (PGC) differentiation to target, we obtained RNA-seq data from NCBI #GSE99350 on WT and *BLIMP1*^-/-^ human iPSCs (hiPSCs) for human PGC-like cells (hPGCLC) induction 14. The early PGC markers including *BLIMP1*, *TFAP2C*, *CD38*, *NANOS3*, and *UTF1* were shown to be upregulated following hPGCLC induction (Fig. S1A). Among these genes, *BLIMP1* has been shown to play a crucial role in hESC differentiation into hPGCLCs 15, 16. *BLIMP1* is known to activate and stabilize a germline transcriptional circuit while repressing a default neuronal differentiation program 15. KO of *BLIMP1* in hESCs has been described to lead to failure of hPGCLC specification and increase the expression of somatic markers during the PGC differentiation 17. Thus, we first knocked out *BLIMP1* in the CT3 hESC line 18 using CRISPR-Cas9 to see whether it is sufficient to block germline differentiation. Genotyping demonstrates that five bases in exon 5 of *BLIMP1* were deleted in both alleles, resulting in a frameshift mutation and loss of BLIMP1 protein expression (Fig. 1A). Then, *BLIMP1^-/-^* hESCs were induced to differentiate into hPGCLCs via embryoid body (EB) formation following a previously reported protocol 19 (Fig. 1B and S1B-C). At day 4 of the differentiation, only WT, but not *BLIMP1^-/-^*, hESCs expressed BLIMP1 (Fig. 1C). Expression of *BLIMP1* and other primordial germ cell (PGC) markers *TFAP2C*, and *SOX17* was remarkably down-regulated in *BLIMP1^-/-^* hESCs at day 4 of the differentiation compared to the WT control (Fig. 1D). Absence of BLIMP1 was also observed in hPGCLCs differentiated from hESCs in monolayer using a recently reported method when examined both by western blotting and immunostaining 20 (Fig. S1D-G). Furthermore, both WT and *BLIMP1^-/-^* hESCs formed teratomas following stochastic differentiation in immunocompromised NOG/SCID mice, thereby demonstrating their pluripotency. The germline marker, DAZL protein (per immunostaining, Fig. 1E) in addition to other germline markers including *VASA*, *STELLA*, and *NANOS3* RNA (per qPCR, Fig. 1F) were all detected in teratomas formed by WT hESCs but virtually absent from those formed by *BLIMP1^-/-^* hESCs. Thus, *BLIMP1* KO blocked both directed and stochastic differentiation of hESCs into hPGCLCs and their derivatives.

### *PAX6* KO in *BLIMP1*^-/-^ hESCs prevents neural differentiation

Next, to select a key gene critical for NPC differentiation, we used a heatmap to analyze the expression of eight NPC markers during hESC differentiation into NPCs. All marker genes were upregulated over time (Fig. S2A). *PAX6* is a determinant for neuroectoderm (NE) specification and is uniformly expressed in the NE differentiated from hESCs 21. *PAX6* KO in hESCs blocks their differentiation into the NE 22 as well as formation of neural organoids 23. Thus, we targeted the *PAX6* gene for KO in our *BLIMP1^-/-^* hESCs in an attempt to abolish their capacity to produce neural lineages. Genotyping showed that 1 base was inserted into exon 1 of *PAX6* in both alleles, leading to a frameshift deletion of the *PAX6* protein (Fig. 2A). We then directly differentiated our *BLIMP1^-/-^*/*PAX6^-/-^* hESCs into neural progenitor cells (NPCs) using either a previously reported protocol 24 (Fig. S2B) or through stochastic differentiation via teratoma formation (Fig. 2B). At D11 of NPC induction, PAX6^+^ cells were detected highly among the WT cells but were negligible among the *BLIMP1^-/-^*/*PAX6^-/-^* cells with flow cytometry (Fig. 2C). At D20 of NPC induction, WT cells displayed neuroepithelial-like morphology with high density whereas *BLIMP1^-/-^*/*PAX6^-/-^* cells appeared to be mesenchymal-like with low density (Fig. S2C). Western blotting also showed that PAX6 and other NPC markers, including NESTIN and SOX2, were present in the WT cells but absent *BLIMP1^-/-^*/*PAX6^-/-^* cells. We also found another NPC marker SOX1 was downregulated remarkably in the *BLIMP1^-/-^*/*PAX6^-/-^* cells compared to the WT cells (Fig. 2D). Immunostaining confirmed the presence of PAX6 and NESTIN in the WT but not *BLIMP1^-/-^*/*PAX6^-/-^* cells (Fig. 2E). Consistent with these findings, neuroepithelial-like cells (derived from the neural ectoderm) were found only in teratomas formed by the WT but not *BLIMP1^-/-^*/*PAX6^-/-^* hESCs, although mesoderm- and endoderm-derived tissues were detected in both groups (Fig. 2F). Immunostaining also showed that the neuronal marker TUJ1 was detected in teratomas formed by the WT, but not *BLIMP1^-/-^*/*PAX6^-/-^*, hESCs (Fig. 2G) while the mesodermal marker SP7 and endodermal marker AFP were detected in teratomas formed by both WT and *BLIMP1^-/-^*/*PAX6^-/-^* hESCs (Fig. S2D and S2E). These results indicate that *PAX6* KO in the *BLIMP1*^-/-^ hESC background could further abolish the potential of cells to undergo neural differentiation.

### Transcriptome profiling of *BLIMP1^-/-^*/*PAX6^-/-^* hESCs shows a loss of differentiation capacity to become gametes and neurons

To test whether pluripotency is retained in the *BLIMP1^-/-^*/*PAX6^-/-^* hESCs, we first confirmed that both WT and *BLIMP1^-/-^*/*PAX6^-/-^* hESCs expressed pluripotency markers NANOG, OCT4, and SOX2 by immunostaining (Fig. 3A). Then, we also performed RNA sequencing (RNA-seq) on WT and *BLIMP1^-/-^*/*PAX6^-/-^* hESCs with or without NPC induction and obtained RNA-seq data from NCBI #GSE99350 on WT and *BLIMP1*^-/-^ hiPSCs with or without PGCLC induction 14. Through principal component analysis (PCA), WT and *BLIMP1*^-/-^ hPSCs were found to be very similar to each other in transcriptome (Fig. S3A), as were WT and *BLIMP1^-/-^*/*PAX6^-/-^* hPSCs (Fig. S3B). However, WT and *BLIMP1*^-/-^ cells exhibited high variance after PGCLC induction (Fig. S3A), as did WT and *BLIMP1^-/-^*/*PAX6^-/-^* cells after NPC induction (Fig. S3B). Volcano plot showed 477 genes were upregulated and 834 genes were downregulated in the *BLIMP1*^-/-^ group compared to the WT control after PGCLC induction (Fig. S3C). The downregulated genes included PGC markers as well as *NANOG* and *PRDM14* which are highly expressed in PGCs 15, whereas some mesodermal and endodermal markers were among those upregulated genes (Fig. 3B).

After NPC induction, 572 genes were downregulated and 389 upregulated in the *BLIMP1^-/-^*/*PAX6^-/-^* group compared to the WT group (Fig. S3D). NPC markers including *PAX6*, *SOX1*, *SOX2*, and *NESTIN* were among those downregulated genes and mesodermal and endodermal markers were among those upregulated genes (Fig. 3C). This was also consistent with our gene ontology (GO) analysis of downregulated (Fig. 3D) and upregulated (Fig. 3F) genes in *BLIMP1^-/-^*/*PAX6^-/-^* cells after NPC induction, which shows that genes in neural-related pathways were downregulated (Fig. 3E) and genes in mesenchyme development pathways were upregulated (Fig. 3G) in the *BLIMP1^-/-^*/*PAX6^-/-^* group compared to the WT group. Together with our results from the teratoma assays, this validates that the DKO of *BLIMP1* and *PAX6* prevents hESCs from germinal and neural differentiation while retaining the potency to generate most other developmental lineages.

### Inducible *BCL2* (*iBCL2*) expression enables robust chimerism of *BLIMP1^-/-^*/*PAX6^-/-^* hESCs with the mouse blastocysts

To unequivocally confirm that *BLIMP1^-/-^*/*PAX6^-/-^* hESCs will not contribute to either germ cells or neural cells during embryogenesis, we employed the human-mouse chimerism assay via microinjection of the mouse blastocyst with the WT and *BLIMP1^-/-^*/*PAX6^-/-^* hESCs. The ENVY hESC line was used for this purpose, which was genetically modified to constitutively express GFP for the injection 25. hPSCs have been described to have low chimerism efficiency with rodent blastocysts due to lower competitiveness and survival than rodent PSCs, which is thought to be caused by activated NFκB and apoptotic signaling pathways in hPSCs 26, 27. Since ectopic expression of the anti-apoptotic gene *BCL2* remarkably increases the survival and chimerism of hESCs with the mouse blastocyst 28, we transduced the ENVY hESCs with lentiviral vectors for *iBCL2* expression upon treatment with DOX (Fig. 4A). Constitutive *BCL2* expression was not chosen as repressed cell death can interrupt the normal development and functions of embryonic cells 29. Indeed, stably transduced iBCL2 lines established from both WT and *BLIMP1^-/-^*/*PAX6^-/-^* hESCs expressed substantially higher levels of BCL2 in the presence of DOX than in its absence per western blotting (Fig. 4B).

Then we injected mouse blastocysts with *iBCL2* hESCs after DOX treatment for 24 hours (h). The injected blastocysts were cultured *in vitro* for 16 h or transplanted immediately into the uterus of a surrogate mouse followed by collection of fetuses at embryonic day 14.5 (E14.5) (Fig. 4C). Immunostaining shows that the apoptotic marker activated caspase 3 (aCasp3) was not detected in the blastocysts injected with *iBCL2* hESCs pretreated with DOX but detected in those without DOX treatment after culturing *in vitro* for 16 h (Fig. 4D). Quantitative PCR (qPCR) at E14.5 showed that human genomic DNA (gDNA) was detected in chimeric fetuses formed by blastocysts injected with both WT and *BLIMP1^-/-^*/*PAX6^-/-^*, DOX-treated *iBCL2* hESCs (hereafter abbreviated as WT and DKO chimeras, respectively) approximating a 10^-4^ dilutions of pure hESC gDNA (Fig. S4A). A human-specific marker STEM121 was detected and overlapped with GFP signal in sections of the chimeras per immunostaining (Fig. S4B). Of E14.5 embryos, 53.3% and 50% contained GFP^+^ human cells for the group injected with DOX-pretreated *iBCL2^+^* WT and *iBCL2^+^*/*BLIMP1^-/-^*/*PAX6^-/-^* hESCs, respectively. In contrast, no GFP^+^ human cells were detected in embryos injected with the *iBCL2^+^* WT hESCs without DOX pretreatment (Table 1). GFP^+^ cells were quantified using ImageJ (NIH, USA) as previously described 30 to calculate the human cell ratio in multiple sections of the WT and DKO chimeras. It ranged from 0.00023 to 0.00093 without significant difference between the two groups (data not shown), consistent with the human gDNA ratio quantified via qPCR (Fig. S4A). These results suggest that, via *iBCL2*, both WT and *BLIMP1^-/-^*/*PAX6^-/-^* hESCs contributed to the chimeric fetuses.

Next, we tracked the human cells in chimeras. We used an antibody specifically against human NANOS3 to detect human germ cells in the gonads of the chimeras. Immunostaining of E14.5 chimeras detected NANOS3 in the gonads of the WT but not DKO chimeras (Fig. 4E and 4F). Similar results were obtained with the antibody Hu-Mito against human mitochondria (Fig. S4C). On the other hand, GFP signal overlapping with the neuronal marker TUJ1 was detected in the spinal cord (Fig. 4G and 4H) and GFP signal overlapping with the NPC marker SOX2 was detected in the brain (Fig. S4D and S4E) of the WT, but not DKO, chimeras. Moreover, the human-specific marker HNA, overlapping with the neuronal marker PAX6, was observed in the brain of the WT, but not DKO, chimeras (Fig. S4F and S4G). In contrast, GFP signals overlapping with the mesodermal marker SP7 and the endodermal marker AFP were detected in the spine (Fig. 4I) and liver (Fig. 4K), respectively, of both WT and DKO chimeras. Significant differences were observed in the ratio of GFP^+^SP7^+^ cell number over the total SP7^+^ cell number or the ratio of GFP^+^AFP^+^ cell number over the total AFP^+^ cell number in the liver, but not the spine, between the WT and DKO chimera groups (Fig. 4J and 4L). These results suggest that DKO specifically prevented hESCs from contributing to germ cells and neural tissues in the chimeras without affecting their capacity to produce meso- and endodermal lineages.

### *Igf1r* KO enables higher human cell contributions into human-mouse chimeras

Igf signaling is essential for the embryonic development and body size 31. Genetic ablation of *Igf1r* in the mouse embryo results in growth retardation and neonatal death 32. A recent study demonstrated that *Igf1r* KO enables robust contribution of rat ESCs into rat-mouse chimeras and prevented reduction of the body size seen with *Igf1r^-/-^* embryos without such chimerization 33. To enhance human cell contribution into human-mouse chimeras, we injected hESCs into *Igf1r^-/-^* mouse blastocysts derived from mating of male and female *Igf1r^+/-^* mice, transplanted the injected blastocysts into the uterus of surrogate mice, and collected embryos at E16 (Fig. 5A). As expected, WT embryos appeared larger than *Igf1r^-/-^* embryos. However, injection of either WT or *BLIMP1^-/-^*/*PAX6^-/-^* hESCs into *Igf1r^-/-^* blastocysts failed to increase the body size and weight, in comparison to non-injected embryos (Fig. 5B-5D), even though higher human gDNA ratios were recorded in *Igf1r^-/-^* than WT embryos injected with WT hESCs (Fig. 5E). This failure may be due to the poor competence of human cells in mouse embryos which is caused by various mechanisms that have been previously described 27.

As seen in chimeras formed by human cell-injected WT mouse embryos (Fig. 4), GFP signals overlapped with the mesodermal marker SP7 and the endodermal marker AFP in all groups (Fig. S5B and S5E). But the GFP^+^ cell ratio was higher in *Igf1r^-/-^* than WT embryos injected with either WT or *BLIMP1^-/-^*/*PAX6^-/-^* hESCs (Fig. S5C and S5F), indicating that human cells showed higher contribution in *Igf1r^-/-^* than WT mouse embryos. Again, human specific NANOS3 in the gonads and HNA signal, overlapping with PAX6, in the brain was only seen in embryos from WT but not *BLIMP1^-/-^*/*PAX6^-/-^* hESCs (Fig. 5F & H). These results suggest that, even with increased contribution of human cells into *Igf1r^-/-^* embryos, *BLIMP1^-/-^*/*PAX6^-/-^* hESCs still only contribute to tissues apart from the CNS and gonads. It is worth noting that the ratio of HNA^+^ cell number/total cell number appeared around 10% (Fig. 5J), much higher than the ratio per qPCR analysis (Fig. S4). However, the images were taken from some human cell-enriched areas. On average, human cells contributed much less in the chimeras.

### Partial rescue of chondrogenesis in *Sox9^+/-^* mice using* BLIMP1^-/-^*/*PAX6^-/-^* hESC-derived mesenchymal stem cells (MSCs)

Recently, we demonstrated that hESC-differentiated MSCs (EMSCs), due to their naturally high BCL2 level, can also form chimera with mouse blastocyst and partially rescue chondrogenesis in mice with heterozygous KO of *Sox9* 34, an essential transcription factor for chondrogenic development 35. Absence of Sox9 in mice causes immature and deformed cartilage and bone development 35. To examine whether *BLIMP1^-/-^*/*PAX6^-/-^* hESCs still have the rescue effect via blastocyst complementation, we first obtained EMSCs through differentiation from WT and *BLIMP1^-/-^*/*PAX6^-/-^* hESCs using the method as reported 34 and verified the MSC features based on the morphology (Fig. S6A) and MSC marker expression (Fig. S6B). Then *Sox9^+/-^* mice were generated via the Cre/loxP system by mating female *Sox9^flox/flox^* mice with male *Prx1/Cre* mice. WT or *BLIMP1^-/-^*/*PAX6^-/-^* EMSCs were injected into *Sox9^+/-^* mouse blastocysts followed by implantation into the uterus of WT surrogate mice immediately. The resultant chimeras were collected at E16 (Fig. 6A). Whole-mount skeletal staining showed retarded skeleton in *Sox9^+/-^* fetuses including decreased chondrogenesis and osteogenesis in thorax and limbs compared to *Sox9^flox/flox^* fetuses, which was largely rescued in chimeric fetuses formed by *Sox9^+/-^* blastocysts injected with WT or *BLIMP1^-/-^*/*PAX6^-/-^* EMSCs (Fig. 6B). Again, *BLIMP1^-/-^*/*PAX6^-/-^* EMSCs failed to contribute to the gonads (Fig. 6C), brain (Fig. 6D) and spinal cord (Fig. S6C) in E16 chimeras.

Next, we let chimeras be born and develop until P1 or P7, which exhibited morphological characteristics and behaviors similar to those of the WT neonates (Fig. 6E and 6H). By analyzing the P1 chimeric neonates, we found that GFP^+^/SP7^+^ and GFP^+^/α-SMA^+^ cells (in yellow) could be found in the spine and skin, respectively (Fig. 6F). Again, *BLIMP1^-/-^*/*PAX6^-/-^* EMSCs failed to contribute to germ cells in the gonads and neuronal cells in the brain and spinal cord, whereas some GFP^+^ cells were found adjacent to these organs (Fig. S6D). Upon qPCR of various organs from P1 chimeric neonates, human gDNA was detected the highest in bone, moderately in skin, heart, kidney, liver, lung, and gut, with little in the gonads and virtually absent from the brain (Fig. 6G). The presence of human gDNA, although at very low levels, in the gonads and brain may be the result of mesenchymal tissues surrounding both organs.

In P7 chimeric neonates, GFP^+^/SP7^+^ cells and GFP^+^/α-SMA^+^ cells (in yellow) were still present in limbs and skins, respectively (Fig. 6I). Human gDNA was also detected in the P7 organs with a pattern like that in the P1 organs but at reduced levels (Fig. 6J), which may be the result of a faster increase in mouse cells than human cells in the chimeras. Additionally, *Sox9^+/-^* mice may also have sufficient chondrogenic cells to compete with the human counterparts in chimeric tissues. Together, these results suggest that *BLIMP1^-/-^*/*PAX6^-/-^* hESC-derived MSCs also partially rescue skeletal defects in *Sox9^+/-^* chimeric fetuses like WT hESC-derived MSCs as we showed previously 34. *BLIMP1^-/-^*/*PAX6^-/-^* human cells neither contributed to germ cells or neural cells nor interfered with chimera development.

## Discussion

In this study we employed a simple, yet effective approach to overcome the ethical challenges associated with human-animal chimeras by specifically preventing the contribution of human neural and reproductive tissues. These challenges arise due to the pluripotency of hPSCs, *i.e.*, their ability to contribute to any tissue in chimeras formed with animal blastocysts 28, 36. This can cause significant ethical concerns regarding the identity of the somehow humanized chimeras and the risk of the possibility that such humanized traits could alter the biological characteristics of the chimeric animals.

To eliminate these concerns, we knocked out *PAX6*, an essential gene for the initial neuroectoderm development from hESCs, which totally prevented hESCs from differentiating to neural cells and contributing to the CNS in chimeras. However, this approach also has drawbacks as it eliminates the possibility of generating potentially therapeutic neurons via chimerism for a variety of neural diseases such as dopaminergic neurons for Parkinson's Disease 37, intracerebral hemorrhage stroke 38 and spinal cord injury 39. Furthermore, *PAX6* is widely regarded as a key transcription factor in eye development, being active in all progenitor cells of the eye including its neuroectoderm, surface ectoderm, and periocular mesenchyme as well as in various differentiated ocular cell types 40. In addition, three members of the PAX transcription factor family including *PAX2*, *PAX4*, and *PAX6* are critical regulators of pancreatic development and differentiation 41. Therefore, deletion of *PAX6* in donor cells may also affect or prevent the genesis of these organs or tissues derived from the donor cells in the host. For this sake, KO of intelligence-specific genes such as CHRM2 42 may be an alternative solution to overcome these ethical concerns.

Another relevant, and perhaps even worse, concern is that human cells contribute to germ cells, carrying over human genes and identity into future generations of the chimeras if poorly controlled, which could, in theory, also potentially spread into the wild. So far, it has not been shown that hPSCs could contribute to germ cells in chimeras formed with animal blastocysts, which may result from some not-yet-identified barrier or the overall low contribution of human cells in chimeras. However, it has been reported that hPSCs can differentiate into PGCLCs i*n vitro* 15, 16 and hPSC-derived hPGCLCs can further develop to oogonia and spermatogonia 43, 44. Thus, all human-animal chimera studies should, in theory, have these safeguards in place in order to meet these ethical safety standards. Here, by knocking out *BLIMP1* in hESCs, we abolished their ability to differentiate into PGCLCs *in vitro* and contribute to germ cells *in vivo* through a variety of assays, thus providing solid evidence that *BLIMP1^-/-^* hESCs meet the necessary ethical standard. Although testis and ovary are essential for reproduction, they are not basic organs directly related to life support. Hence, there is no urgent demand for organogenesis of testis and ovary via chimerism. Furthermore, *BLIMP1* plays a unique role in adaptive immunity by driving plasma cell formation in B cells and regulating effector functions in T cells, and also influences innate immunity by controlling dendritic cell maturation. It functions by competing with other transcription factors and by recruiting chromatin-modifying enzymes in dose- and context-dependent manners 45. Thus, *BLIMP1^-/-^* hESCs should not be used for generating human hematopoietic cells in host animals.

In this study, we knocked out two relevant genes in donor hESCs using CRISPR/Cas9 to prevent the cell differentiation into germline and neural cells within the host. To mitigate potential effects of these KOs on early developmental processes that require these genes, they may, instead, be deleted using an inducible system.

Apart from ethical concerns, the major histocompatibility (MHC) as well as minor histocompatibility (MiHC) of a donor organ and its recipient are also important for organogenesis as their mismatch will cause varying degree of immune rejection 46. Use of patient-derived iPSCs, if used for organogenesis in a chimera, can reduce this concern. However, xenogeneic glycoproteins, *e.g.*, α-1,3-galactose, NeuGc, SDa, can also trigger serious immune rejection in a host receiving transplantation of tissues or organs carrying these xenogeneic proteins 46. This could also occur upon transplantation of a chimera-derived organ to a human patient even with an HLA match. Thus, KO of these genes in a host animal is probably also necessary to reduce the contamination of chimera-derived organs by xenogeneic glycoproteins 47-49.

In our study, we employed *Igf1r^-/-^* mice to enhance the chimeric efficiency of donor hESCs. Although the human DNA content was increased in chimeras derived from *Igf1r^-/-^* mouse blastocysts, the body size of these chimeras did not differ from those derived from the WT blastocysts. This outcome may reflect the inherently low chimeric efficiency between hESCs and mouse blastocysts, even with *Igf1r* KO, due to interspecies incompatibility, as reported by other groups 50, 51. In addition, we used cartilage compensation as a proof-of-concept for organogenesis in a chimera based on our recent findings that EMSCs can form chimera with the mouse blastocyst 34. However, it was an incomplete recovery of the cartilage, indicating that *BLIMP1^-/-^*/*PAX6^-/-^* cells may not behave totally in the same way as WT cells, which will require further investigation. Additionally, cartilage is not a tissue in high demand for transplantation and heterozygous deletion of *Sox9* is not sufficient for human MSC derivatives to dominate or take over chondrogenic tissues. Thus, a key test for blastocyst complementation will be complete KO of a gene(s) for the development of a life-essential organ or tissue such as the kidney, liver, and islet tissue. Furthermore, larger animals such as pigs or monkeys would be more appropriate species than the mouse for genesis of human organs via chimerism. Therefore, future work will be necessary to determine whether our findings extend to the generation of life-essential organs in larger animals.

Although we addressed two major ethical concerns in this study, there are more challenges to be resolved in chimera-based organogenesis. For example, it remains unclear exactly how much the chimerism models actually reflect real organogenesis in humans, whether induced expression of *BCL2* at hESC stage would affect the differentiation and morphogenesis of their derivatives or even induce tumorigenesis. Another big challenge will be the removal of host-derived supporting cells such as stromal cells, vascular cells, neural cells, tissue-resident immune cells from human organs and replacing them with human counterparts. Moreover, chimerism efficiency was still quite low, especially at the later developmental stages. It is not only determined by the survival of hPSCs in an animal blastocyst, but also by competition and selection of the donor cells in the host. The innate immunity/NFκB pathway has been found to be a conserved gatekeeper for a host embryo to eliminate less-fit donor cells during interspecies chimerism, and inhibition of the pathway and use of naïve, instead of primed, hPSCs can overcome this gatekeeper effect 26, 27. Regardless of these remaining concerns, this study has taken one important step towards the realization of human-animal chimeras as a method to study human organogenesis and potential future clinical applications.

## Materials & Methods

### Ethics statement

This study strictly followed the International Society for Stem Cell Research guidelines for studies on hESCs 52, 53 and the ethics protocol #BSERE19-APP026-FHS-M02 approved by the University of Macau Panel on Research Ethics which allowed human-mouse chimeras to develop to term with the neonates secured and terminated within two weeks. Animal experiments in this study followed the amended version of the animal use protocol #UMARE-030-2019 approved by the University of Macau Animal Research Ethics Sub-panel.

### Mice

Male and female ICR (CD-1) mice, raised in the University of Macau Animal Research Core, were used to produce WT blastocysts, and male *Prrx1^Cre^* mice and female *Sox9^flox/flox^* mice, purchased from the Jackson Laboratory, were crossed to produce *Sox9^+/-^* blastocysts. *Igf1r^-/-^* mouse blastocysts were generated by mating *Igf1r^+/-^* male and female mice purchased from the European Mouse Mutant Archive. The blastocysts were used for microinjection with donor cells. ICR female mice were used for surrogacy of the injected blastocysts. All mice were maintained in the animal research core and tested to be free of specific pathogens.

### hESC culture and MSC generation

The ENVY (GFP^+^) 25 and CT3 hESC lines 18 were used in this study. hESCs were cultured in mTeSR1 medium and passaged every 5-7 days 54. EMSCs were generated by inducing hESCs to differentiate into MSC via neural crest cells following the reported protocol 55. Specifically, hESCs were seeded in mTeSR1 medium for overnight attachment. Cells were applied with neural crest induction medium consisting of 1 μM dorsomorphin (Selleck), 1 μM CHIR99021 (Selleck), 10 μM SB431542 (Selleck), 10 ng/ml bFGF (Thermo), and 22.5 ng/ml sodium heparin (Sigma) in Essential 6 (E6) medium (Gibco) for 15 days. Cells were split at a ratio of 1:6 upon reaching full confluence. Then complete MSC medium was applied for further 10 days induction to complete full EMSCs derivation.

Derived MSCs were cultured in complete MSC medium containing 20% fetal bovine serum, 1× NEAA and 1× GlutaMax in α-MEM (Gibco BRL, Grand Island, NY, USA) at 37 ℃ with 5% CO_2_. MSCs were passaged every 3-5 days when confluency reached more than 90%, using 1× TrypLE for 5-min. incubation followed by seeding of 15% of resuspended cells in a new culture plate. The cells were verified for typical MSC markers via flow cytometry and trilineage differentiation and used in this study before they reached passage 5.

### Generation of *BLIMP1* and *PAX6* DKO cell lines

By using previously reported sgRNA sequences (Table S1) 16, 56, we generated two CRISPR-Cas9 expression vectors to target *BLIMP1* and *PAX6*, respectively. The *BLIMP1*-targeting vector was transfected into hESCs. After 48 h of puromycin selection for ENVY hESCs or cell sorting for CT3 hESCs, single-cell cloning was performed to identify clones with successful *BLIMP1*-KO which was verified via sequencing of the target gDNA following PCR amplification with specific oligos (Table S1). Then, *PAX6*-targeting vector was transfected into the hESCs with *BLIMP1* KO. After 48 h of puromycin selection for ENVY hESCs or cell sorting for CT3 hESCs, single cell cloning was performed to seek for clones with successful *PAX6*-KO which was verified via sequencing of the target gDNA following PCR amplification with specific oligos (Table S2). The resultant cell lines were confirmed for the DKO of both *BLIMP1* and *PAX6*.

### Generation of DOX-inducible *BCL2*-OE hESCs

A full-length *BCL2* fragment was cloned from EF1a-BCL2-IRES-eGFP-IRES-PuroR vector (Addgene) and then the construct was inserted into BamHI- and NdeI-digested Ptight-TYR-IRES-EGFP-PuroR vector (Addgene) by using the In-fusion Cloning Kit (Takara). For viral vector packaging, PMD2G, PCMVR8.74, and Ptight-BLC2-IRES-EGFP-PuroR or pCAG-tet-ON-NeoR vector (Addgene) were transfected together into 293T cells by using lipofectamine 3000. Viral supernatant was collected 24-48 h post transfection and added to culture of WT and *BLIMP1^-/-^*/*PAX6^-/-^* hESCs together with 1:1000 polybrene. 48 h later, the cells were selected using puromycin (500 ng/ml) and neomycin (800 mg/ml) for two weeks. Stably selected cells were confirmed for DOX-inducible expression of BCL2.

### Differentiation of hESCs into hPGCLCs

By following a reported protocol 19, hESCs were dissociated into single cells using TrypLE. Briefly, 200,000 dissociated hESCs were seeded in Matrigel-coated 12-well-plates in 1 ml of the iMeLC media per well to generate incipient mesoderm-like cells. After 24 h, 3000 dissociated incipient mesoderm-like cells were seeded into ultra-low cell attachment U-bottom 96-well plates per well with 200 μL of the hPGCLC media. After 4 days, hPGCLCs were generated and confirmed for PGC markers.

### Differentiation of hESCs into NPCs

By using a reported protocol 24, hESCs were treated with 4 μM CHIR99021, 3 μM SB431542, and 0.1 μM Compound E in a neural induction medium containing Advanced DMEM/F12:Neurobasal (1:1) (Gibco), 1×N2, 1×B27, 1% GlutaMAX, 5 μg/mL BSA and 10 ng/mL hLIF (Millipore) for 7 days. Then, the resultant NPCs were cultured with 3 μM CHIR99021 and 2 μM SB431542 in the neural induction medium for expansion.

### Teratoma formation assay

As reported 57, a mixture of 50 μl DPBS containing 10^6^ hESCs and 50 μl Matrigel was grafted into the thigh of each hindlimb of a male NOD/SCID mouse around 6 weeks of age. Tumors were collected 2 months after the injection and fixed in 4% paraformaldehyde (PFA) overnight followed by embedding in paraffin. For H&E staining and immunostaining, the tumors were sectioned at 5 μm thick.

### Transcriptomic profiling and analysis

As reported 58, total RNA of WT and *BLIMP1^-/-^*/*PAX6^-/-^* hESCs with or without NPC induction were isolated by TRIzol reagent (Invitrogen) and RNA-seq was conducted via Illumina NovaSeq 6000 by following the manufacturer's instructions. Raw data of WT and *BLIMP1*^-/-^ hESCs with or without PGCLC induction (GSE99350) were downloaded for read-count analysis. Qualified reads for above samples were aligned to the Homo sapiens reference genome (GRCh38.p12) via STAR software (v2.7.0f). Genes with zero count in all samples were removed before downstream analysis. The EdgeR (v3.40.2) and DESeq2 (v1.38.3) package in R (v4.2.2) was applied to perform PCA and differentially expressed genes (DEGs) analysis. Among them, in edgeR analysis, genes with log2 fold change(log2FC) > 2 and p value < 0.01 were considered as DEGs and in DESeq2 analysis, the threshold of DEGs was log2FC > 1 and adjusted *P* value < 0.05. Gene ontology (GO) term enrichment and dotplots was performed by using Cluster Profiler (v4.6.2) and enrichplot (v1.18.3). Heatmaps were generated by pheatmap (v1.0.12) and ggplots package (v3.4.0).

### Blastocyst microinjection

Superovulation, mating, and blastocyst retrieval were performed as described previously 59. Briefly as reported 34, female ICR mice (3~4 weeks old) were superovulated through i.p. injection with 5 IU of pregnant mare serum gonadotropin (PMSG). After 48 h, they were injected i.p. with 5 IU of human chorionic gonadotropin (hCG) and mated with male mice. Plugs were checked the next morning, which is designated as 0.5-day post coitum (dpc). Females with plugs were used as surrogate host for embryo implantation and were euthanized by CO_2_ exposure at 3.5 dpc. Each uterine horn was flushed with 1 mL M2 medium. The embryos that possess obvious blastocoel were picked up using mouth pipette. Cells that need to be injected were dissociated in M2 medium. 10-15 cells loaded into a micropipette (20 μm inner diameter) were injected into the cavity of each mouse blastocyst. The injected blastocysts were kept in warm M2 medium before being transferred to *in vitro* culture or to surrogate hosts.

### Embryo implantation and sample collection

3-6 months ICR female mice were mated with vasectomized adult male mice. Plugs were checked the next morning, which is designated as 0.5 dpc. Adult ICR female mice in estrus were mated with vasectomized adult male in 1:1 pairing to achieve pseudopregnancy. Females with plugs were used as surrogate host for embryo implantation. Pseudopregnant ICR mice at 2.5 dpc were anesthetized and each mouse was implanted with 10-12 injected blastocysts loaded into a glass pipette and transferred to each of the two uterine horns of the mouse. The implantation was finished within 20 min per surrogate and the operated animals were kept warm via heating until they woke up from anesthetization. Fetuses or P1 and P7 neonates were euthanized and fixed for subsequent analyses.

### DNA quantification of chimeras

E14.5 chimeric fetuses and 2-day-old neonates were minced with scissors and tissue pieces were then frozen using liquid nitrogen and smashed. DNA was extracted using the DNA Extraction Kit (Beyotime) based on the manufacturer's instructions. DNA concentration was determined using NanoDrop. A standard curve was made using various ratios of mouse and hESC DNA in 100 ng DNA in total. Human gDNA was detected using iTaq Universal SYBR Green (Bio-Rad) on a CFX96 Touch RT-qPCR system (Bio-Rad). The human specific gene used in this study is *ARHGAP11B* 60.

### Immunostaining

Tissue sections or cultured cells, fixed in 4% PFA, were permeabilized with 0.5% Triton X-100 in PBS for 20 min. Samples were then blocked with 5% BSA in PBS for 1 h and incubated overnight at 4 °C with the first antibody. After washing three times using PBS including 0.1% Tween20 (PBS-T), the samples were incubated at room temperature for 1 h with secondary antibody, which was washed three times using PBS-T again. Cell nuclei were stained with 4',6-diamidino-2-phenylindole (DAPI) for 5 min. Samples were imaged on a Carl Zeiss Axio Observer microscope for individual imaging or SpinSR 10 spinning disk confocal microscopy for large-scale imaging (Olympus Life Science, Japan). The proteins detected via immunostaining were as follows: BLIMP1, DAZL, NANOS3, PAX6, NESTIN, TUJ1, aCASP3, BCL2, STEM121, GFP-FITC, SOX2, SP7, and AFP, and human mitochondria were stained using the antibody Hu-Mito (antibody information listed in Table S3). For quantification of human cell contribution to a target cell type, at least 3 chimeras and 3 slides per chimera per group were visualized. Cells positive for a specific fluorochrome were quantified using ImageJ (NIH, USA) as previously described 30. The ratio of human cell contribution was calculated as % of the number of cells positive for both GFP and the immunostained target cell marker over the total number of cells positive for the marker per observed view, unless stated otherwise.

### Western blotting

10% SDS-polyacrylamide gel was used to resolve cellular proteins. The proteins were transferred to a polyvinylidene difluoride (PVDF) membrane (Bio-Rad) using the Semi-dry Transference (Bio-Rad). PVDF membrane was then blocked in PBS-T containing 5% skim milk for 1 h. The membrane was incubated overnight at 4 °C with a first antibody (antibody information listed in Table S3). After washing three times with PBST, the membrane was incubated at room temperature for 1 h with the secondary antibody, which was washed three times using PBS-T again. The membrane was rinsed in an enhanced chemiluminescence (ECL) (Bio-Rad) substrate and imaged using the Western Blotting Detection System (Bio-Rad).

### RNA extraction and reverse transcription quantitative PCR (RT-qPCR)

Total RNA was extracted from human cells or teratomas using the Trizol reagent (Thermo). The RNA concentration was determined using NanoDrop. cDNA was generated with the cDNA Reverse Transcription Kit (Takara). RT-qPCR was performed using iTaq Universal SYBR Green on a CFX96 Touch RT-qPCR system using primers as listed in Table S2.

### Data analysis

Data analyses were performed by using GraphPad Prism 9. One-way ANOVA analysis was used for multiple group comparisons and two-tailed Student's *t*-test was used for two-group comparisons. *P < 0.05 was considered statistically significant. The results are displayed as the mean ± standard error (SE).

## Supplementary Material

Supplementary figures.

## Figures and Tables

**Figure 1:**
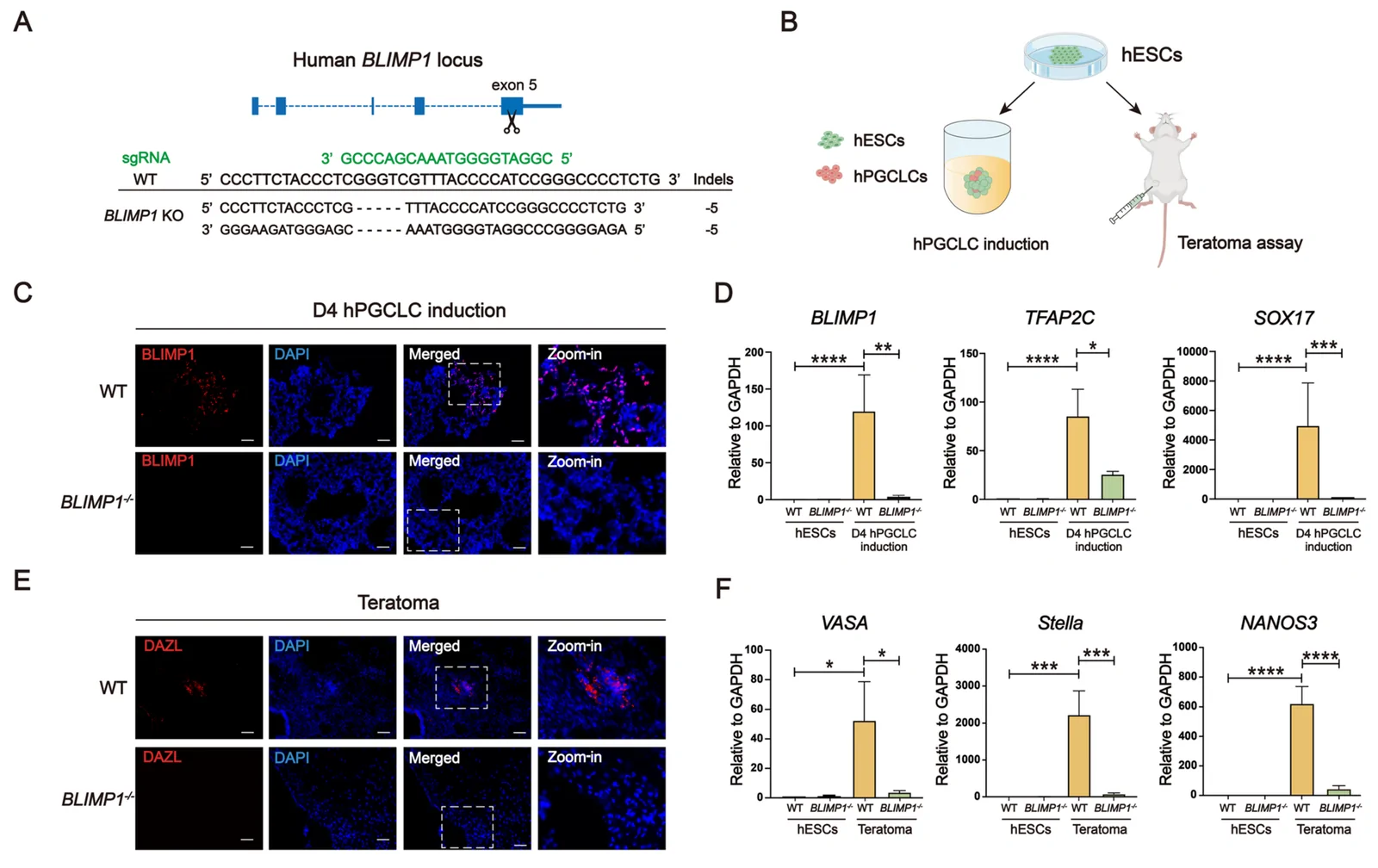
***BLIMP1***** KO prevents hESCs from differentiating into germ cells.** (**A**) Strategy and genotyping for *BLIMP1* KO. (**B**) Schematic for directed differentiation of hESCs into hPGCLCs *in vitro* and random differentiation of hESCs to form teratoma *in vivo*. (**C**) Immunostaining for BLIMP1 on WT and *BLIMP1*^-/-^ hESCs at day 4 (D4) of hPGCLC induction. Scale bar, 25 μm. (**D**) Quantitative PCR (qPCR) to detect germline markers in WT and *BLIMP1*^-/-^ hESCs at D4 of hPGCLC induction (n = 3). (**E**) Immunostaining for the germline marker DAZL in teratomas formed by WT and *BLIMP1*^-/-^ hESCs. Scale bar, 25 μm. (**F**) qPCR to detect germline markers in teratomas formed by WT and *BLIMP1*^-/-^ hESCs (n = 3). *P < 0.05, **P < 0.01, ***P < 0.001, ****P < 0.0001.

**Figure 2:**
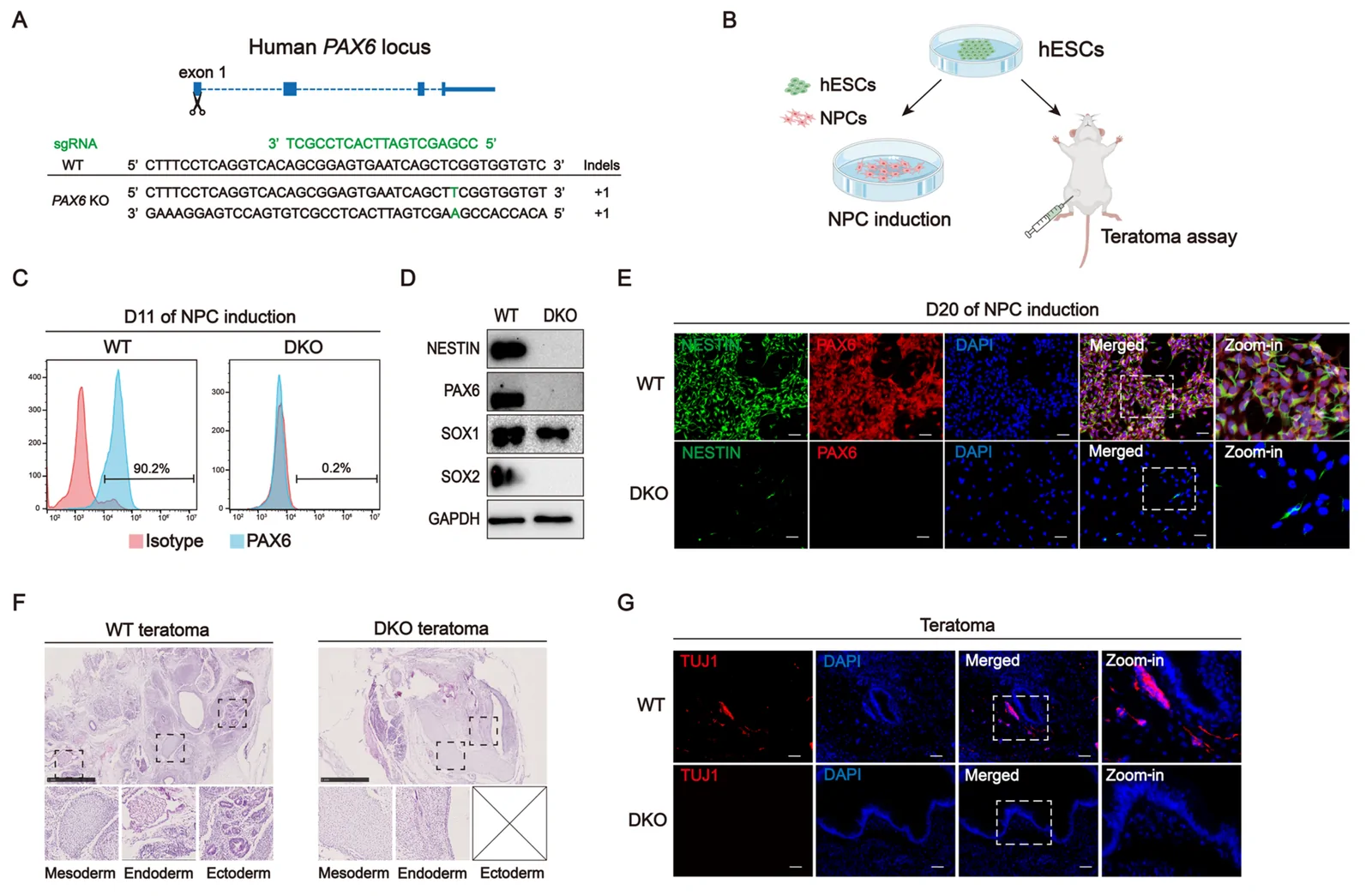
***PAX6* KO prevents *BLIMP1^-/-^* hESCs from neural differentiation.** (**A**) Strategy and genotyping for *PAX6* KO. (**B**) Schematic for directed differentiation of *BLIMP1^-/-^*/*PAX6^-/-^* hESCs into NPCs *in vitro* and random differentiation of the *BLIMP1^-/-^*/*PAX6^-/-^* hESCs to form teratomas *in vivo*. (**C**) Flow cytometry for PAX6 in WT and *BLIMP1^-/-^*/*PAX6^-/-^* hESCs at D11 of NPC induction. (**D & E**) Western blotting (**D**) and immunostaining (**E**) for NPC markers in WT and *BLIMP1^-/-^*/*PAX6^-/-^* hESCs at D20 of NPC induction. Scale bar, 25 μm. (**F**) Hematoxylin and eosin staining on sections from teratomas formed by WT and *BLIMP1^-/-^*/*PAX6^-/-^* hESCs. Scale bar, 250 μm. (**G**) Immunostaining for the neuronal marker TUJ1 on sections from the above teratomas. Scale bar, 25 μm.

**Figure 3:**
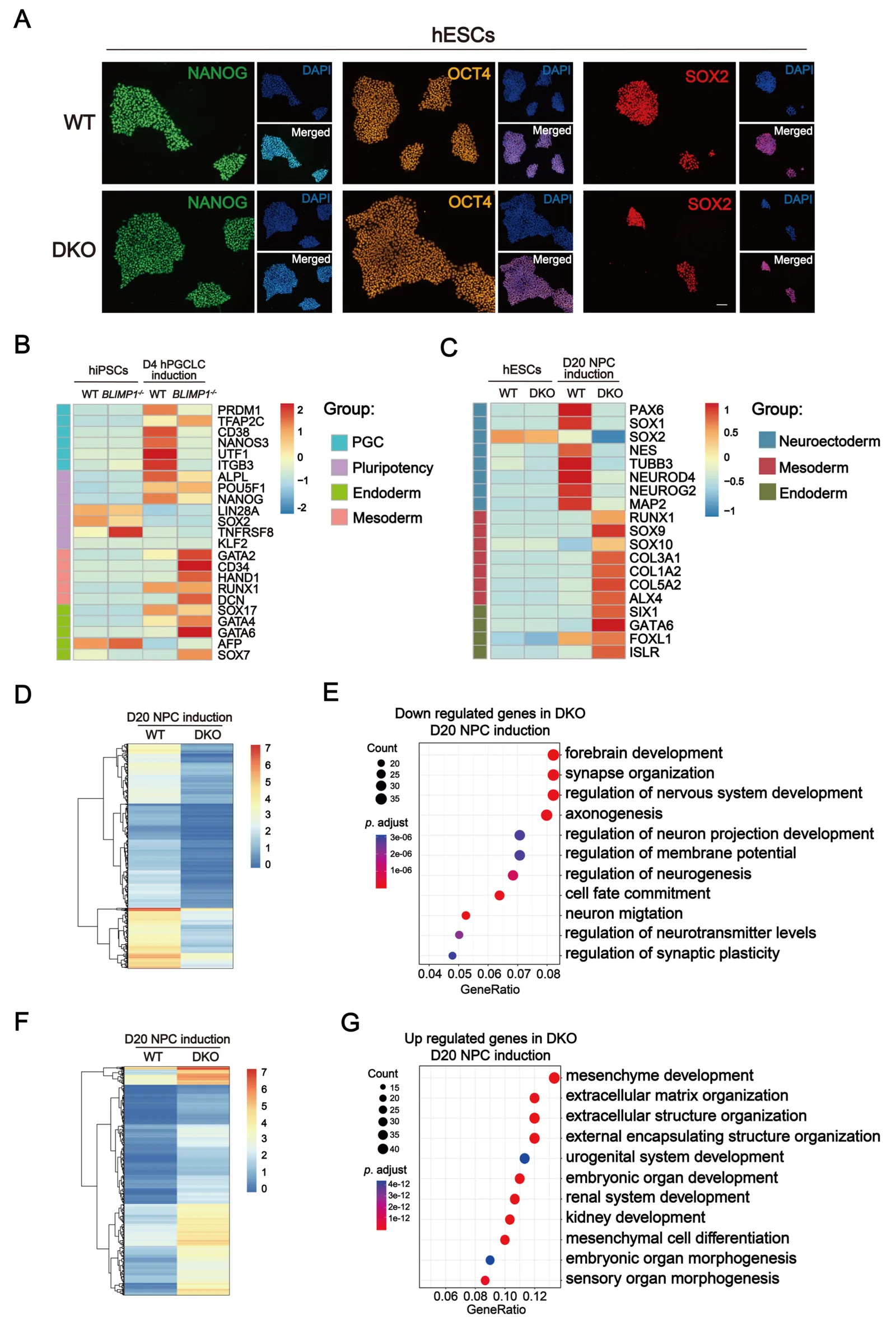
** Transcriptomic comparison of WT and *BLIMP1^-/-^*/*PAX6^-/-^* hESCs and derivatives.** (**A**) Immunostaining for pluripotency markers in WT and *BLIMP1^-/-^*/*PAX6^-/-^* hESCs. Scale bar, 25 μm. (**B & C**) Heatmap for designated marker gene expression in WT and *BLIMP1*^-/-^ hiPSCs with or without PGCLC induction (**B**) and WT and *BLIMP1^-/-^*/*PAX6^-/-^* hESCs with or without NPC induction (**C**). (**D & F**) Heatmap for downregulated (**D**) and upregulated (**F**) genes in *BLIMP1^-/-^*/*PAX6^-/-^* cells compared to WT cells after NPC induction. (**E & G**) GO analysis for top terms associated with downregulated (**E**) and upregulated (**G**) genes in *BLIMP1^-/-^*/*PAX6^-/-^* cells versus WT cells after NPC induction.

**Figure 4:**
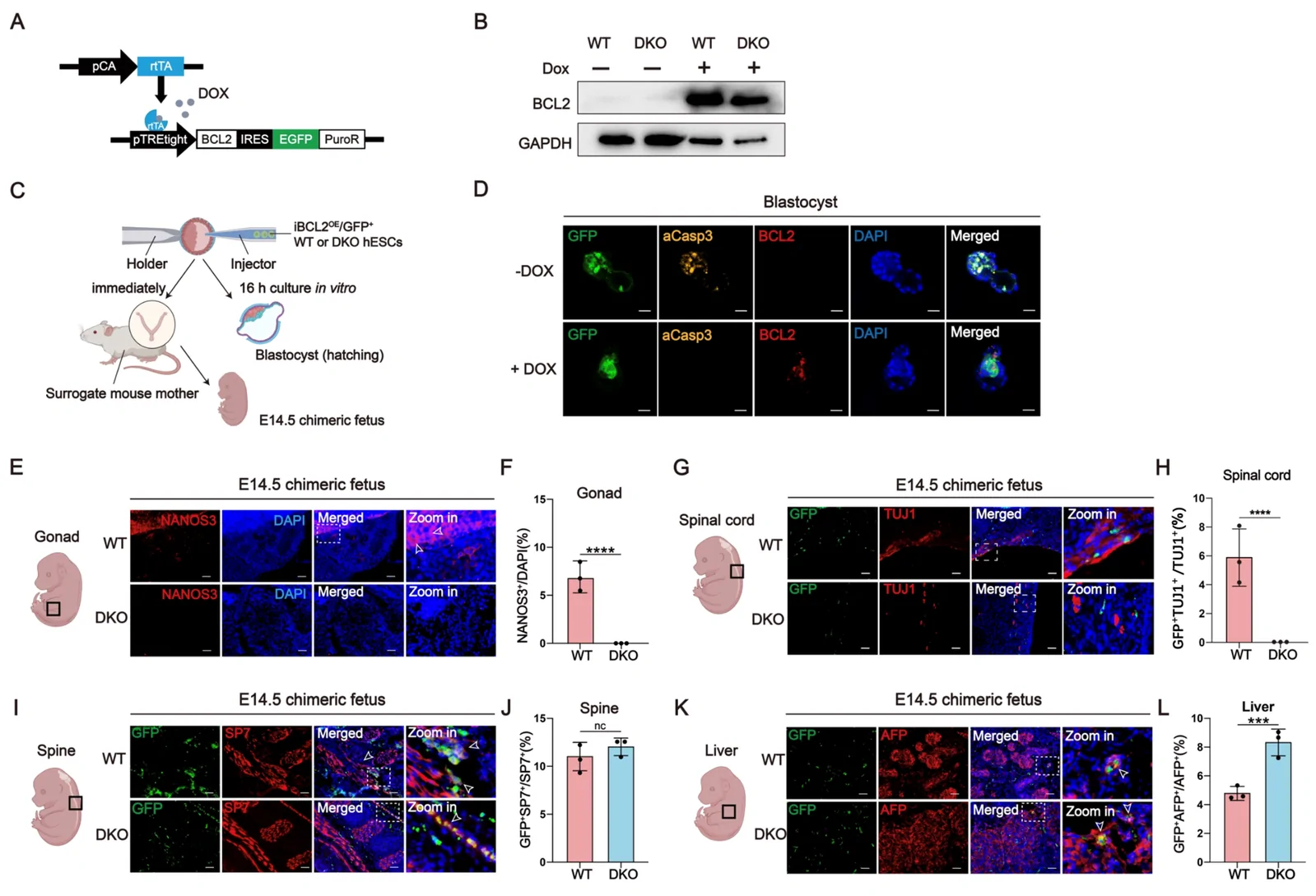
***iBCL2* allows robust chimerism of *BLIMP1^-/-^*/*PAX6^-/-^* hESCs with the mouse blastocyst without gametal and neural contributions.** (**A**) Schematic for a DOX-inducible BCL2 (*iBCL2*) expression construct. (**B**) Western blotting for BCL2 expression in both WT and *BLIMP1^-/-^*/*PAX6^-/-^ iBCL2* hESCs after DOX treatment for 24 h. (**C**) Schematic for blastocyst injection, implantation into the uterus of a surrogate mouse or *in vitro* culture followed by sample collections. (**D**) Immunostaining for BCL2 and aCasp3 in injected mouse blastocysts cultured *in vitro* for 16 h. (**E-L**) Immunostaining and quantification for NANOS3 in the gonad (**E & F**), TUJ1 in the spinal cord (**G & H**), SP7 in the spine (**I & J**), and AFP in the liver (**K & L**) in both E14.5 WT and DKO chimeras. Scale bar, 25 μm. Quantification method: the NANOS3^+^/DAPI^+^ cell ratio stands for the ratio of the NANOS3^+^ cell number over the total DAPI^+^ cell number in observed views of the gonad, and likewise for GFP^+^TUJ1^+^/TUJ1^+^ ratio in the brain, GFP^+^SP7^+^/SP7^+^ ratio in the spine, and GFP^+^AFP^+^/AFP^+^ ratio in the liver. Scale bar, 25 μm. *P < 0.05, **P < 0.01, ***P < 0.001, ****P < 0.0001.

**Figure 5:**
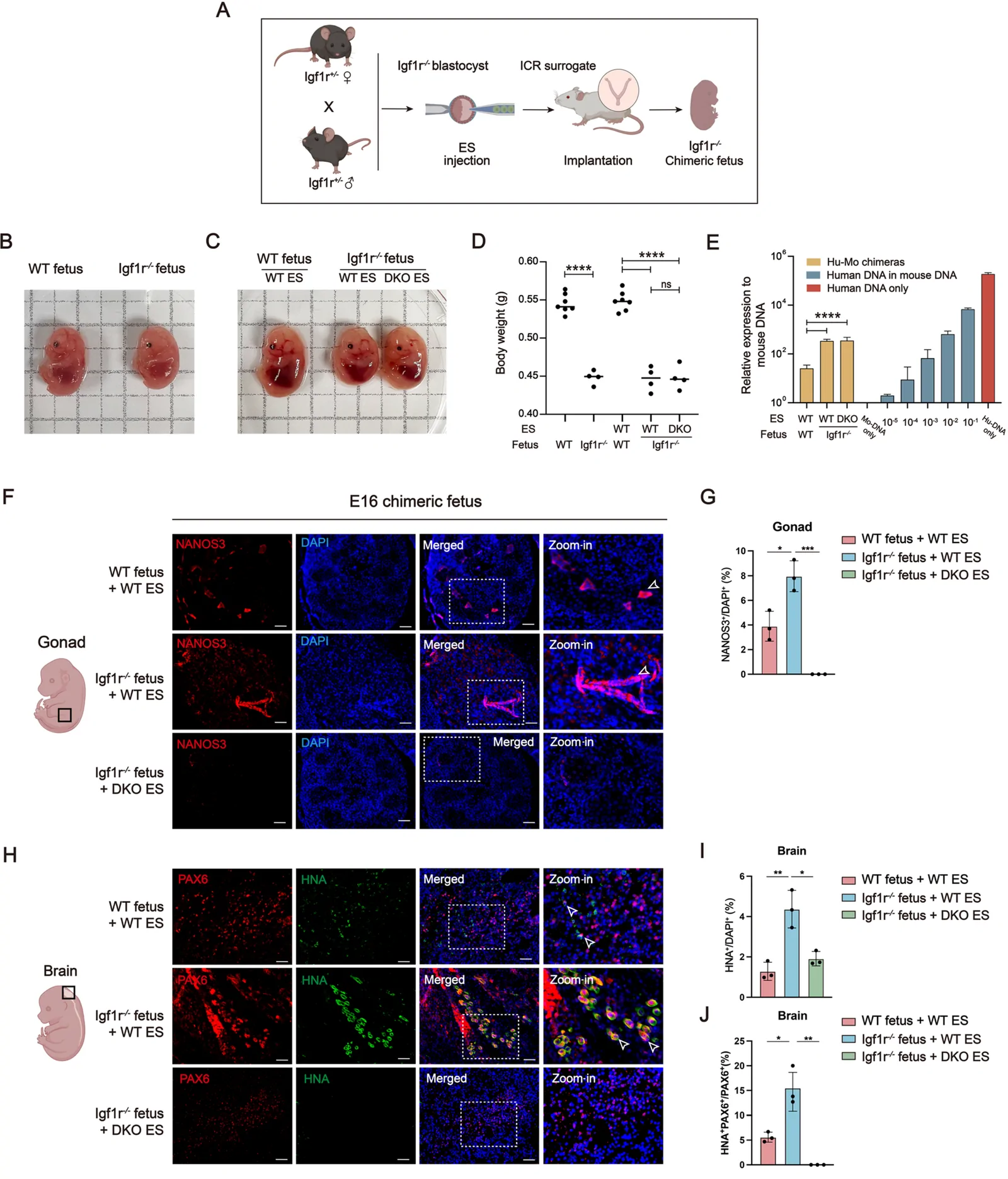
***Igf1r* KO allows increased human cell chimerism with the mouse embryo without neural and gametal contributions.** (**A**) Schematic for generation of *Igf1r^-/-^* mouse blastocysts, injection with hESCs (ES), and uterine implantation. (B) Representative images of E16 fetuses derived from WT or* Igf1r^-/-^* mice. (**C-E**) Representative images (C), body weight (D), and human gDNA quantification (E) of E16 fetuses derived from WT or* Igf1r^-/-^* mouse blastocysts injected with WT or *BLIMP1^-/-^*/*PAX6^-/-^* hESCs. (**F**) Immunostaining for the human germ cell marker NANOS3 in the gonads in E16 chimeras. (**G**) Quantification for the NANOS3^+^/DAPI^+^ cell ratio in the gonad. (**H**) Immunostaining for the neural marker PAX6 and human-specific marker HNA in the brain in E16 chimeras. (**I**) HNA^+^DAPI^+^/DAPI^+^ cell ratio in the brain. (**J**) HNA^+^PAX6^+^/PAX6^+^ cell ratio in the brain. Scale bar, 25 μm. *P < 0.05, **P < 0.01, ***P < 0.001, ****P < 0.0001.

**Figure 6:**
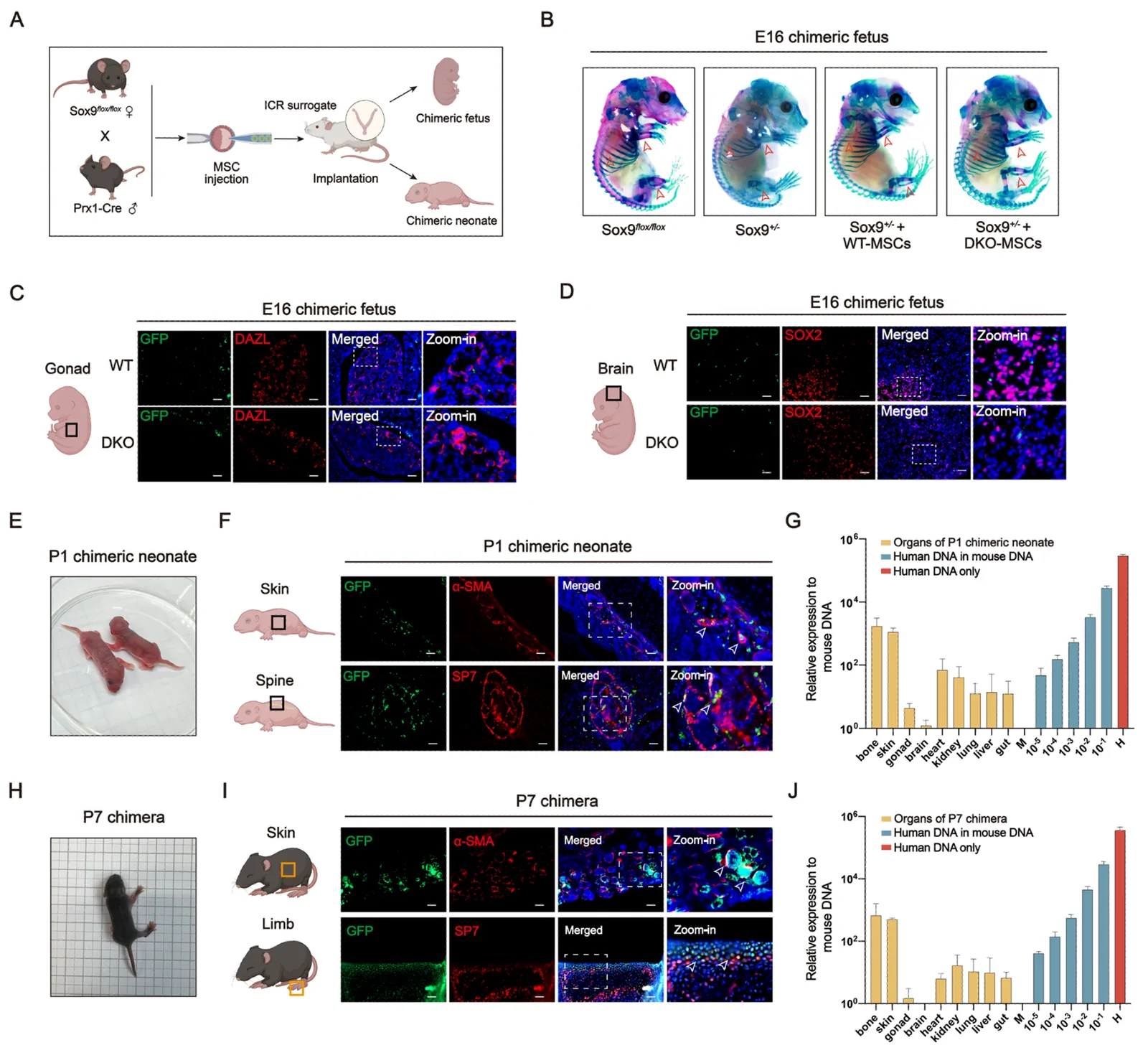
***BLIMP1^-/-^*/*PAX6^-/-^* hESC-derived MSCs partially rescue chondrogenesis in** ***Sox9^+/-^*** **mice and human gDNA detection in P1 and P7 chimeric neonates.** (**A**) Schematic for *Sox9^+/-^* blastocyst generation, blastocyst injection, uterine implantation in a surrogate mouse, and sample collections. (**B**) Skeletal images of E16 fetuses derived from *Sox9^flox/flox^* ,* Sox9^+/-^* blastocysts, and *Sox9^+/-^* blastocyst injected with WT and *BLIMP1^-/-^*/*PAX6^-/-^* EMSCs. (**C-D**) Immunostaining for the germline marker DAZL in the gonads (**C**), the neural marker SOX2 in the brain (**D**) in E16 chimeras. Scale bar, 25 μm. (**E**) Representative image for P1 chimeric neonates. (**F**) Detection of GFP^+^ and SP7^+^ cells in the spine, GFP^+^ and aSMA^+^ cells in the skin in P1 chimeric neonates. Scale bar, 25 μm. (**G**) Human gDNA quantification in organs from P1 chimeric neonates. (**H**) A representative image of P7 chimeras. (**I**) Detection of GFP^+^/SP7^+^ cells in a limb and GFP^+^/aSMA^+^ cells in skin of P7 chimeric neonates. Scale bar, 25 μm. (**J**) Human gDNA quantification in organs of the P7 chimeric neonates.

**Table 1:** Summary of efficiency for chimerism between the mouse blastocyst and injected hESCs with various genetic modifications*.

hESCs for injection	DOX induction	# of transplanted embryos	# of collected embryos (%)	# of chimeras (%)
*GFP*^+^/*iBCL2^+^* hESCs	Yes	84	15 (17.8)	8 (53.3)
*GFP*^+^/*iBCL2^+^*/*BLIMP1^-/-^*/*PAX6^-/-^* hESCs	Yes	81	14 (17.3)	7 (50)
*GFP*^+^/*iBCL2^+^* hESCs	No	42	8 (19.0)	0

*10-15 cells were injected per blastocyst. Embryos were collected at E14.5.
