# Supplementary material for: Human-Mouse Chimerism for Organogenesis Without Neural or Germline Contributions
Source: Int J Biol Sci. 2026 Sep 3;22(14):7948–62. doi: 10.7150/ijbs.119609 (PMC13618233; doi:10.7150/ijbs.119609)
Supplement: Supplementary file 1 [file ijbsv22p7948s1.pdf]

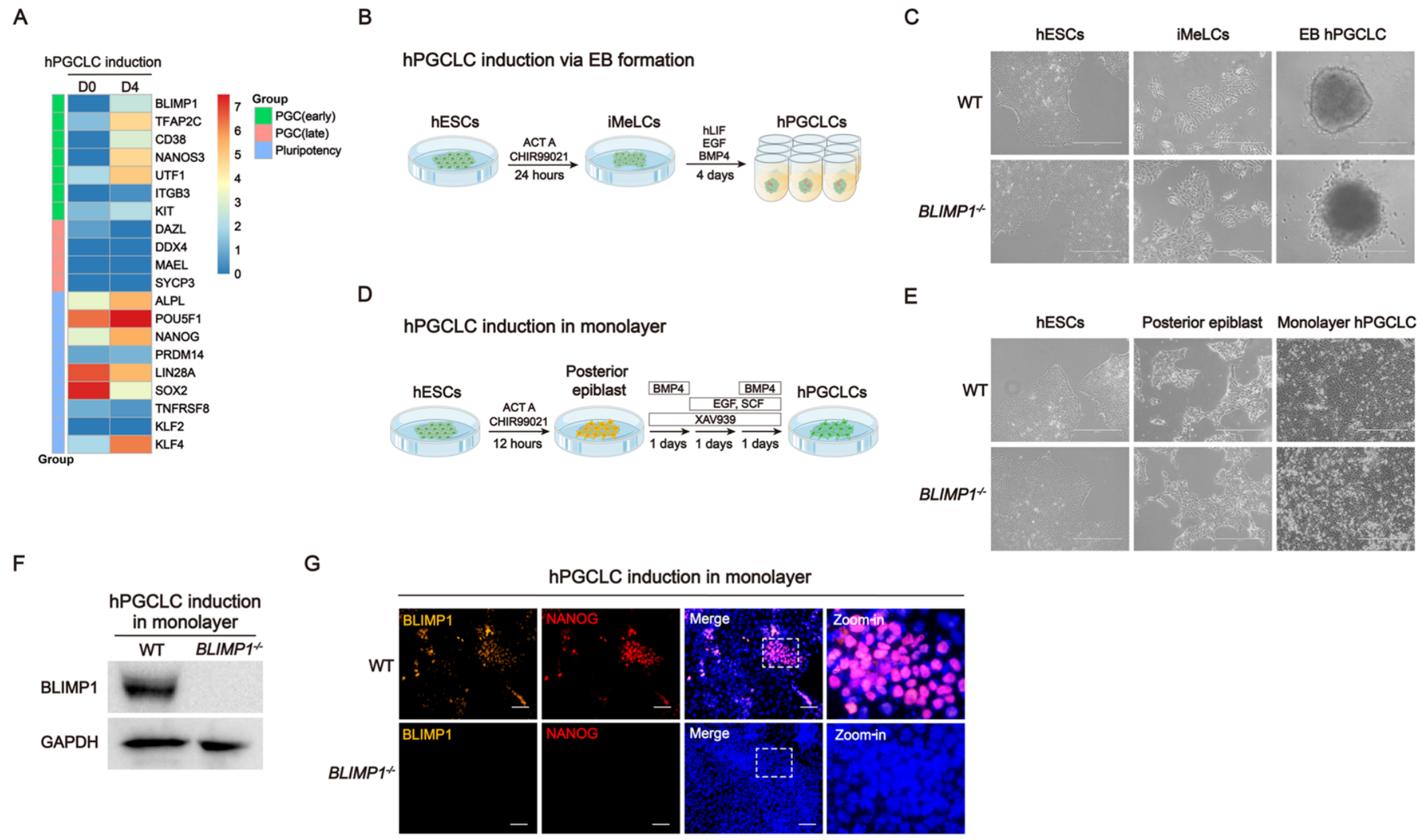

Figure S1. Schematic graph and representative images for PGCLC induction from WT and *BLIMP1$^{-/-}$* hESCs through embryoid body (EB) or monolayer. (A) Heatmap for expression of markers for early PGCs, late PGCs, and pluripotency in hiPSCs at D0 and D4 of PGCLC induction. **(B & C)** Schematic graph (**B**) and representative images (**C**) for hESCs under PGCLC induction through EB. Scale bar, 400 µm. **(D & E)** Schematic graph (**D**) and representative images (**E**) for PGCLC differentiation through monolayer. Scale bar, 400 µm. **(F)** Western blotting for BLIMP1 in PGCLCs induced from hESCs through monolayer. **(G)** Immunostaining for PGC markers BLIMP1 and NANOG in PGCLCs induced from hESCs through monolayer. Scale bar, 25 µm.

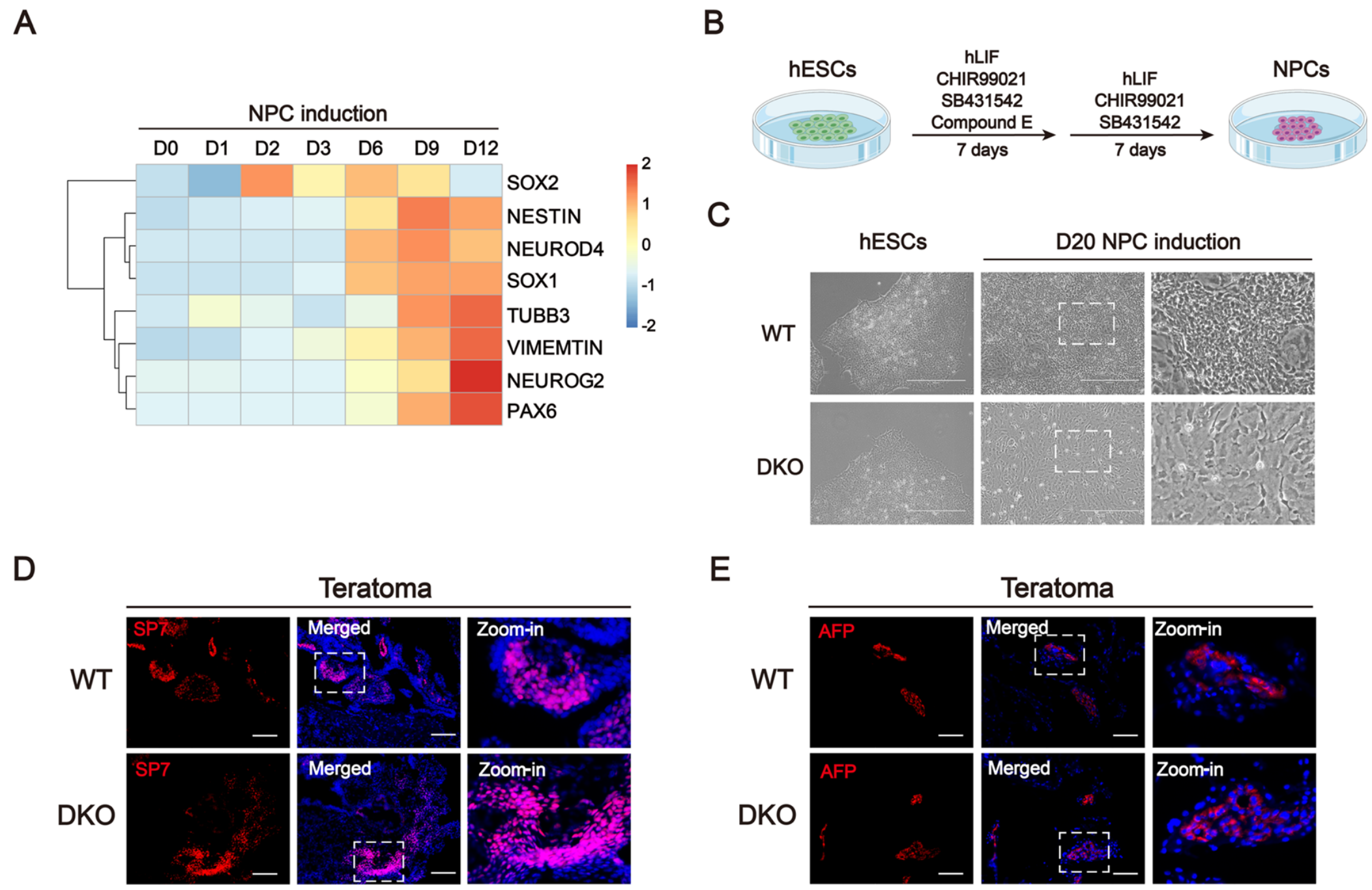

Figure S2. NPC induction and teratoma formation by WT and *BLIMP1*$^{-/-}$/*PAX6*$^{-/-}$ hESCs. (A) Heatmap for expression of NPC markers in hESCs at various days of NPC induction. **(B & C)** Schematic graph (**B**) and representative images (**C**) for hESCs differentiation into NPCs. Scale bar, 400 µm. **(D & E)** Immunostaining for a mesodermal marker SP7 and endodermal marker AFP in teratomas formed by WT and *BLIMP1*$^{-/-}$/*PAX6*$^{-/-}$ hESCs. Scale bar, 25 µm.

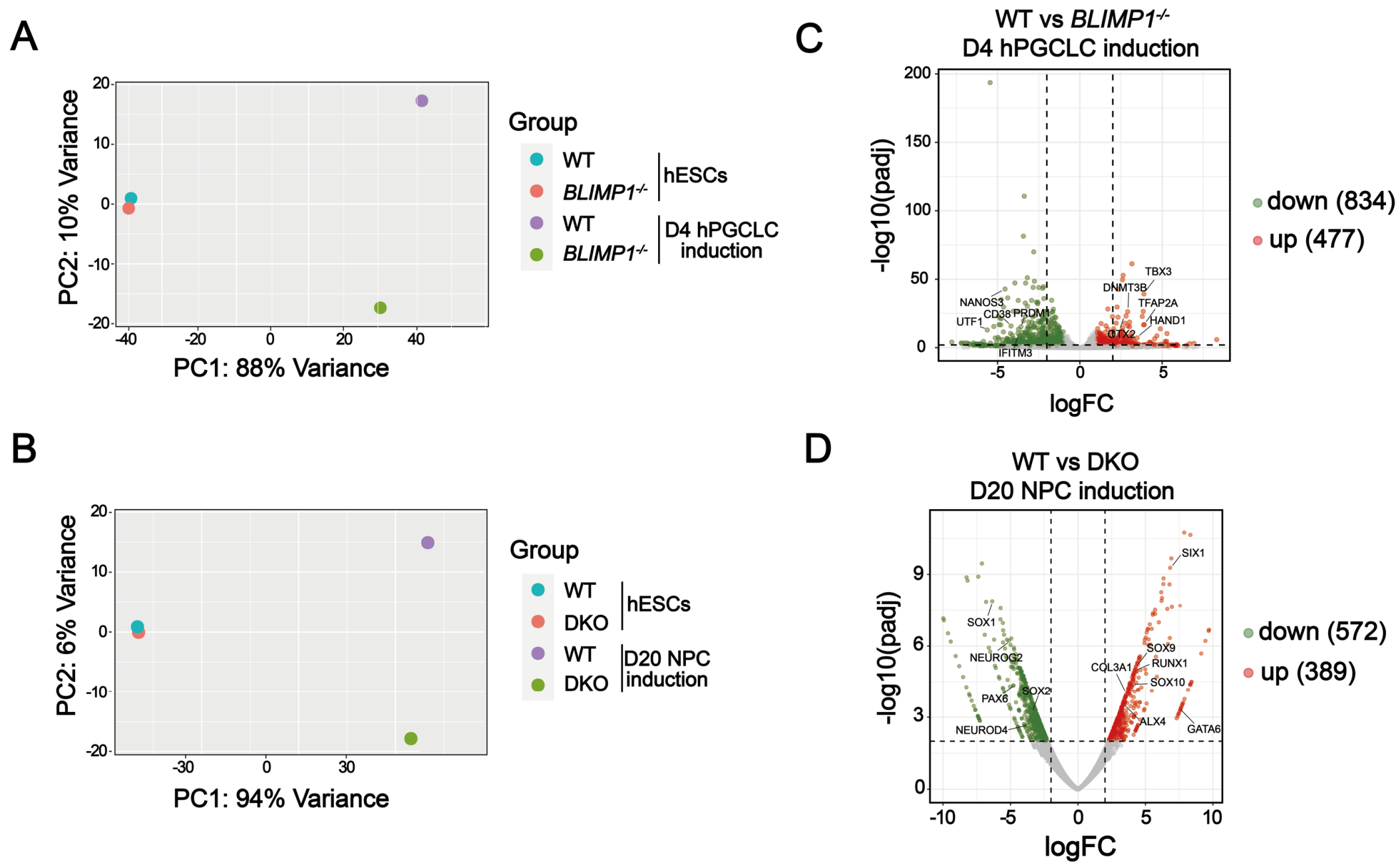

Figure S3. Transcriptomic comparison of WT and *BLIMP1*$^{-/-}$/*PAX6*$^{-/-}$ hESCs and derivatives via PCA and volcano plot analyses. (**A-B**) PCA for WT and *BLIMP1*$^{-/-}$ hiPSCs with or without PGCLC induction (**A**) and WT and *BLIMP1*$^{-/-}$/*PAX6*$^{-/-}$ hESCs with or without NPC induction (**B**). (**C-D**) Volcano plot for differentially expressed genes (DEGs) in WT and *BLIMP1*$^{-/-}$ cells after PGCLC induction (**C**) and in WT and *BLIMP1*$^{-/-}$/*PAX6*$^{-/-}$ cells after NPC induction (**D**).

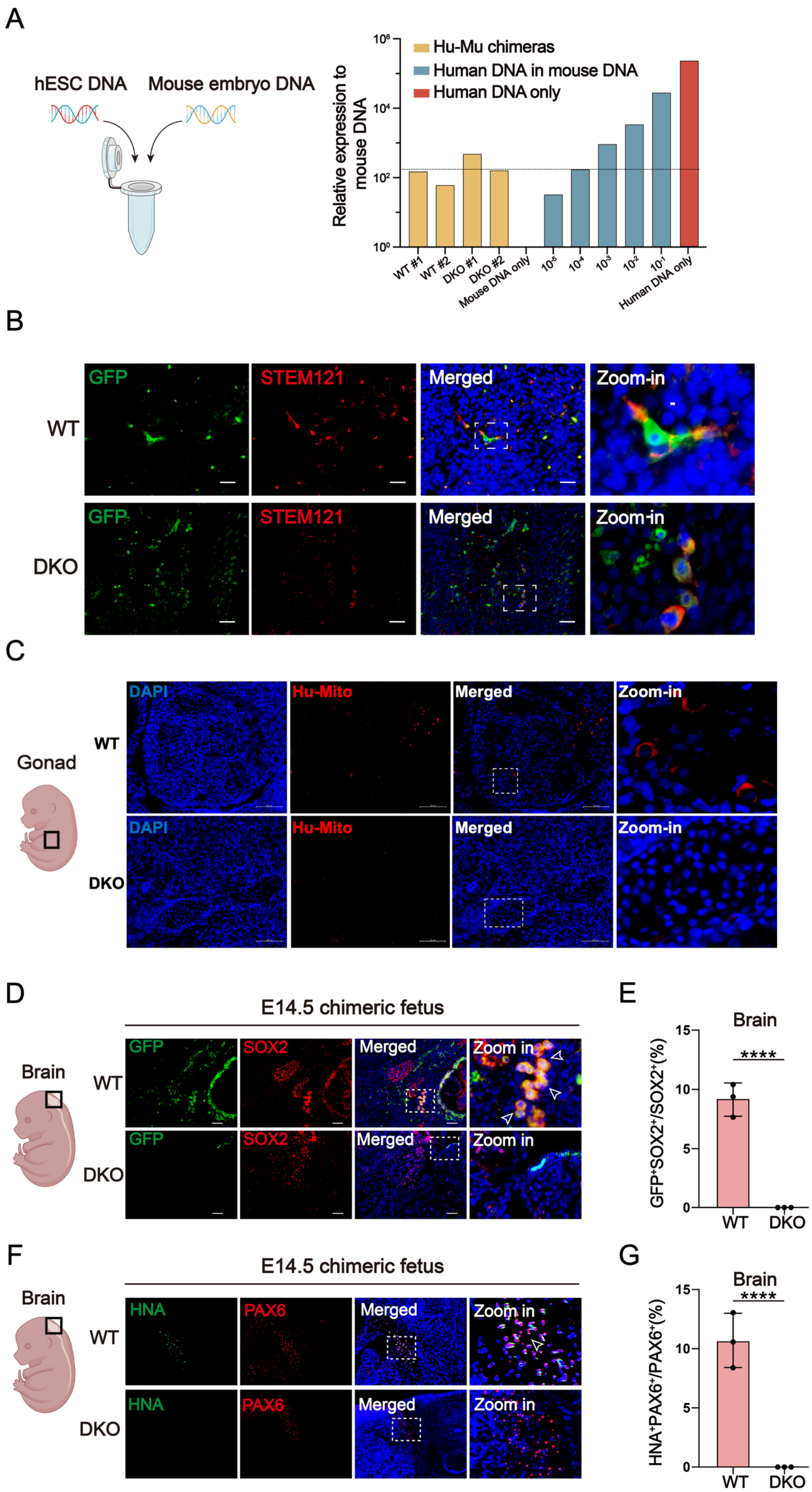
A
hESC DNA
Mouse embryo DNA
Hu-Mu chimeras
Human DNA in mouse DNA
Human DNA only
Relative expression to mouse DNA
WT #1
WT #2
DKO #1
DKO #2
Mouse DNA only
Human DNA only
B
WT
DKO
GFP
STEM121
Merged
Zoom-in
C
Gonad
DAPI
Hu-Mito
D
E14.5 chimeric fetus
Brain
SOX2
Zoom in
E
GFP+SOX2+/SOX2+(%)
****
F
HNA
PAX6
G
HNA+PAX6+/PAX6+(%)

Figure S4. Human gDNA and human cell detection in E14.5 chimeras formed by mouse blastocysts injected with both WT and *BLIMP1*$^{-/-}$/*PAX6*$^{-/-}$, DOX-treated *iBCL2* hESCs. (**A)** Human gDNA quantification in E14.5 chimeras. (**B**) Immunostaining for a human-specific marker STEM121 in the E14.5 chimeras. Scale bar, 25 µm. (**C**) Immunostaining for human mitochondria, using the antibody Hu-Mito, in the gonad of both E14.5 WT and DKO chimeras. Scale bar, 100 µm. (**D**) Immunostaining for SOX2 in the brain in both E14.5 WT and DKO chimeras. (**E**) Quantification for the $GFP^{+}SOX2^{+}/SOX2^{+}$ cell ratio in observed views of the brain. (**F**) Immunostaining for PAX6 and HNA in the brain in both E14.5 WT and DKO chimeras. (**G**) Quantification for the $HNA^{+}PAX6^{+}/PAX6^{+}$ cell ratio in observed views of the brain. Scale bar, 25 µm. *$P < 0.05$, **$P < 0.01$, ***$P < 0.001$, ****$P < 0.0001$.

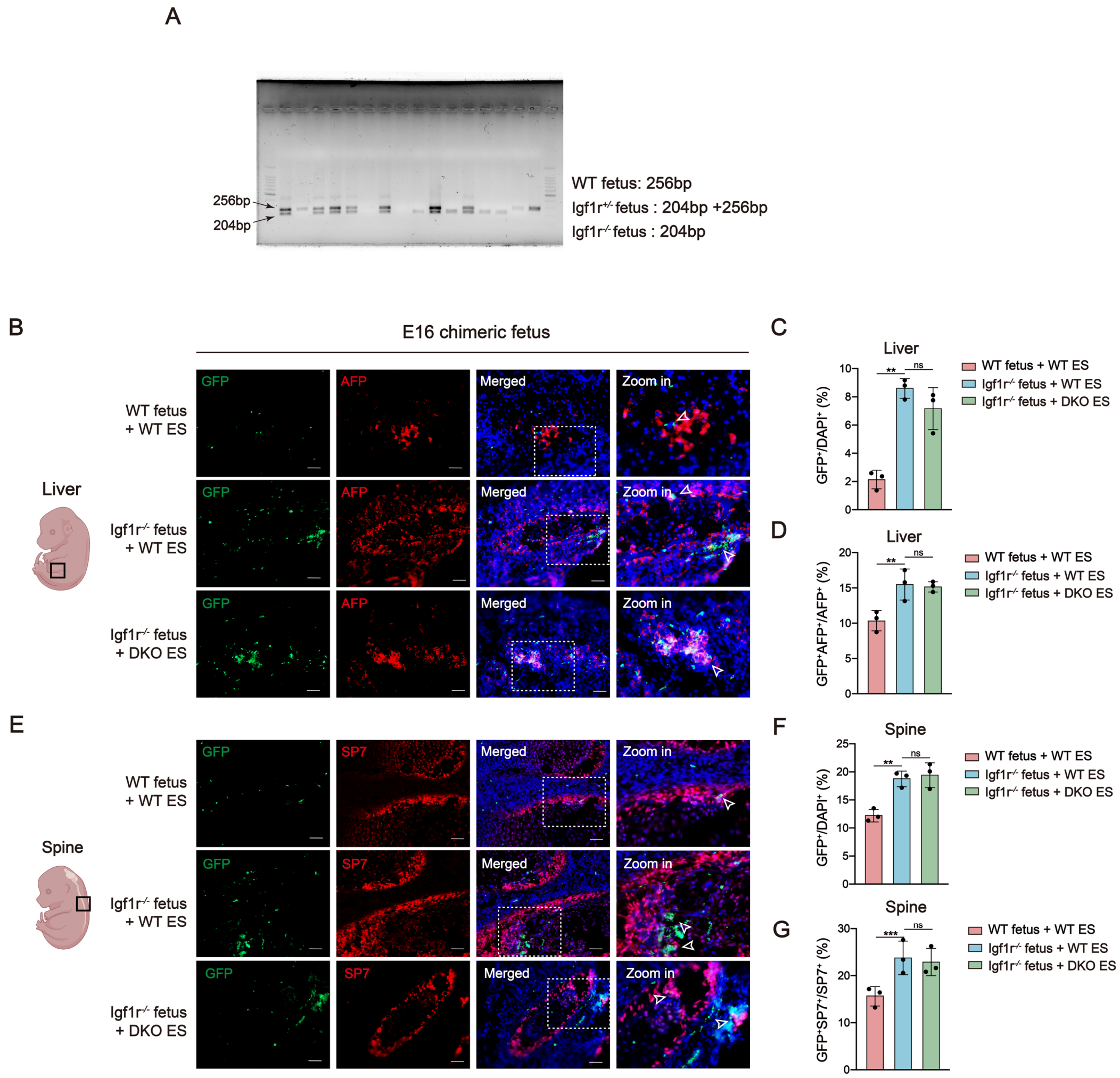

Figure S5. Genotyping for *Igf1r*$^{(-/-)}$ chimeras and immunostaining for human cell contributions into E16 chimeras. (**A**) Genotyping for *Igf1r* KO chimeras. (**B**) Immunostaining for the endodermal marker AFP in the liver in E16 chimeras. (**C**) Quantification for the GFP$^+$DAPI$^+$/DAPI$^+$ cell ratio in the liver. (**D**) GFP$^+$AFP$^+$/AFP$^+$ cell ratio in the liver. (**E**) Immunostaining for the mesodermal marker SP7 in the spine in E16 chimeras. (**F**) GFP$^+$DAPI$^+$/DAPI$^+$ cell ratio in the spine. (**G**) GFP$^+$SP7$^+$/SP7$^+$ cell ratio in the spine. Scale bar, 25 µm. *$P < 0.05$, **$P < 0.01$, ***$P < 0.001$, ****$P < 0.0001$.

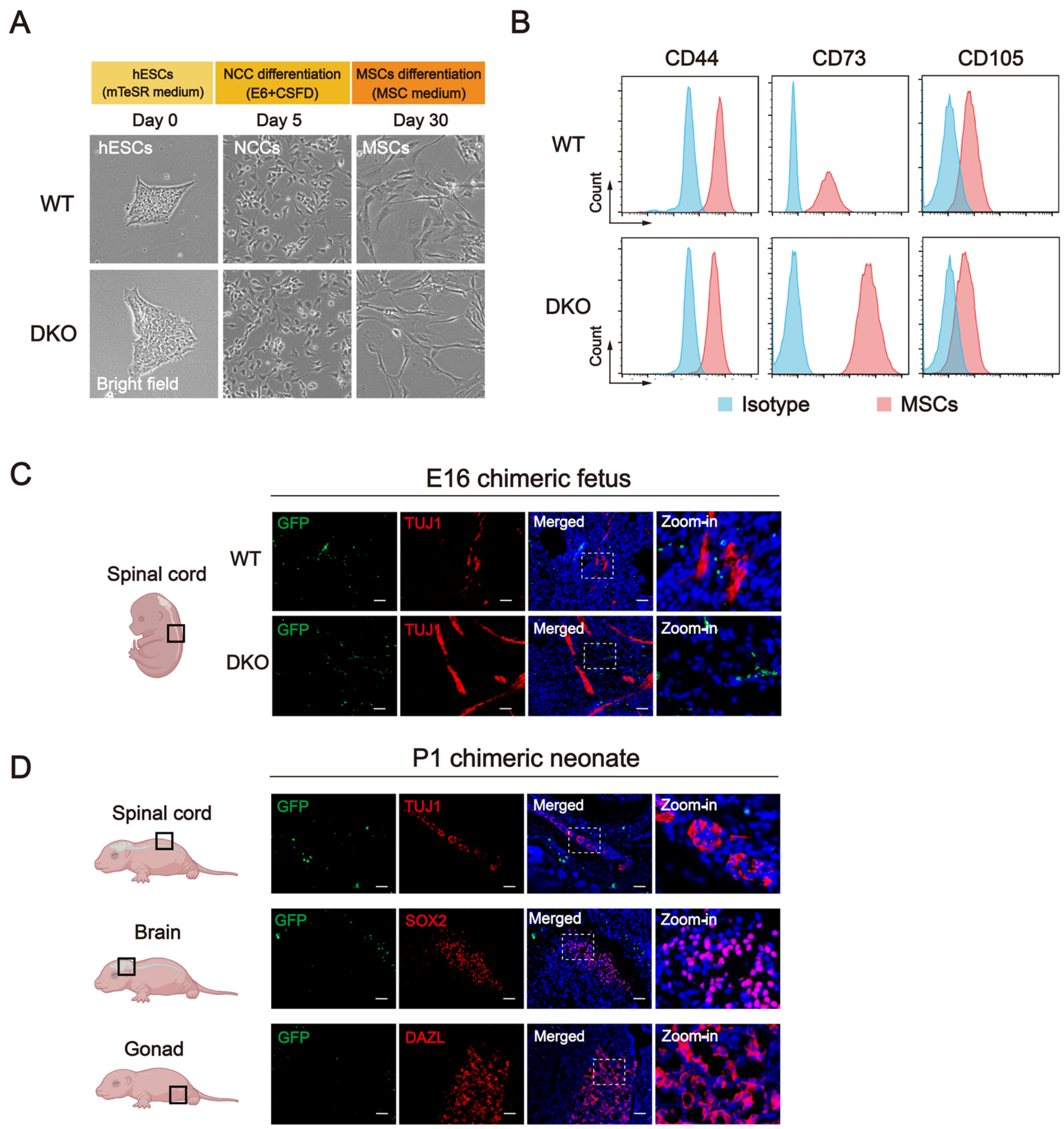

Figure S6. WT and *BLIMP1*$^{-/-}$/*PAX6*$^{-/-}$ hESC-derived MSCs contributed to mesenchymal tissues but not the CNS and gonads in *Sox9*$^{+/-}$ mice. (**A**) Schematic graph and representative images for hESC differentiation into MSCs (EMSCs). (**B**) Flow cytometry analysis for MSC markers. (**C**) Immunostaining for the neural markers TUJ1 in the spinal cord in both E16 WT and *BLIMP1*$^{-/-}$/*PAX6*$^{-/-}$ chimeras. Scale bar, 25 µm. (**D**) Immunostaining for the germline marker DAZL in the gonads, neural markers SOX2 in the brain and TUJ1 in the spinal cord in P1 chimeric neonates. Scale bar, 25 µm.
